# Choc hémorragique suite à une ponction biopsie rénale (PBR): à propos d'un cas

**DOI:** 10.11604/pamj.2015.22.115.6292

**Published:** 2015-10-12

**Authors:** Jalal El Hammoumi, Brahim Boukatta, Nawfal Houari, Abderrahim Elbouazzaoui, Hicham Sbai, Nabil Kanjaa

**Affiliations:** 1 Service de Réanimation Polyvalente A4, CHU Hassan II, Fès, Maroc

**Keywords:** Insuffisance rénale, ponction transcutanée, complication, choc hémorragique, Renal failure, transcutaneous puncture, complication, hemorrhagic shock

## Abstract

La ponction biopsie rénale (PBR) est un examen indispensable en néphrologie mais à risque de complications graves surtout hémorragique. Nous rapportons l'observation d'un patient âgé de 27 ans ayant bénéficié d'une PBR pour un diagnostic étiologique d'une insuffisance rénale aigue, l’évolution après la biopsie a été marquée par l'installation d'un choc hémorragique d'où la prise en charge en réanimation avec une néphrectomie d'hémostase.

## Introduction

La ponction biopsie rénale (PBR) est un examen indispensable en néphrologie permettant un apport de diagnostic histologique des maladies rénales parenchymateuses; mais à risque de complications graves. Les incidents sont essentiellement hémorragiques. Nous rapportons ici un cas de choc hémorragique suite à une ponction biopsie rénale écho-guidée faite dans le cadre du diagnostic étiologique d'une insuffisance rénale aiguë.

## Patient et observation

Mr K.M âgé de 27 ans, a été hospitalisé au service de néphrologie pour la prise en charge d'une insuffisance rénale rapidement progressive. L'examen à l'admission a trouvé un patient conscient stable sur le plan hémodynamique et respiratoire avec une pression artérielle à 120/70 mmHg et une fréquence cardiaque à 75 bpm. Sur le plan biologique, l'hémoglobine était à 9,6g/dl, le TP à 60%, l'urée à 1,46 g/l et la créatininémie à 117 mg/l. Le reste du bilan était sans particularité. Dans le cadre d'une recherche étiologique une ponction biopsie rénale à gauche a été réalisée sous contrôle échographique. L’évolution, 24 h après le geste a été marquée par l'apparition d'une douleur au niveau de l'hypochondre gauche, une pâleur, une tachycardie avec une polypnée. Les conjonctives étaient décolorées. La pression artérielle était à 100/60 mmHg, la fréquence cardiaque à 120 bpm et la fréquence respiratoire à 24 cycles/min. Sur le plan biologique, l'hémoglobine était à 8g/dl, le TP à 45%, le taux de plaquettes était normal. Une échographie abdominale a mis en évidence un épanchement cloisonnée à contenu échogène au niveau de l'espace spléno-rénal.

**Figure 1:**
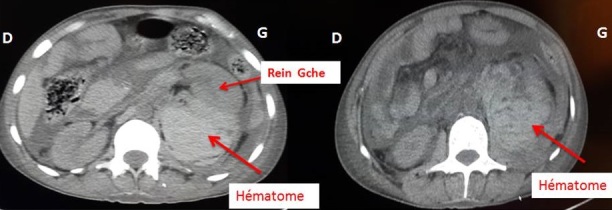
Scanner abdominal, coupes transversales (sans injection du produis de contraste), montrant un volumineux hématome occupant l'espace rétropéritonéale refoulant le rein gauche en avant

**Figure 2:**
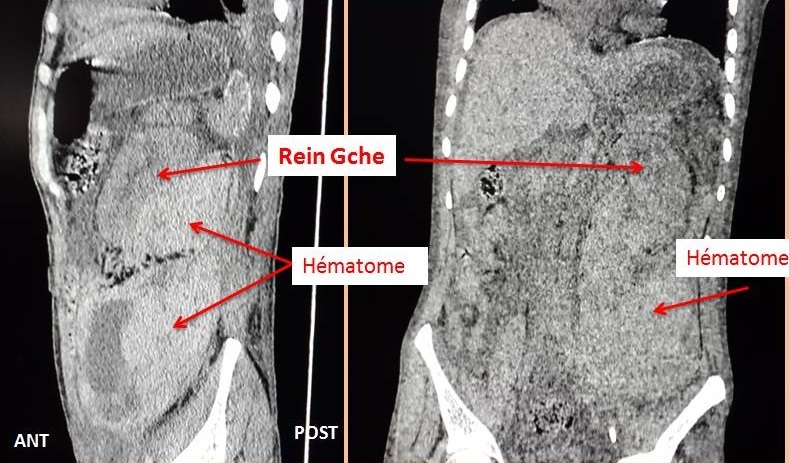
Scanner abdominal, coupe sagittale et coronale (sans injection du produis de contraste), montrant un volumineux hématome péri-rénal postérieur gauche diffusant au niveau de la région pelvienne homolatérale

**Figure 3:**
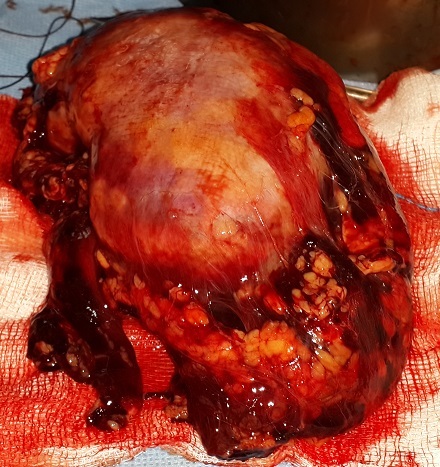
Pièce opératoire après néphrectomie (rein gauche)

Le scanner abdominal sans injection a montré un volumineux hématome péri-rénal gauche compressif, diffusant au niveau de la région pelvienne homolatérale (Figure 1, Figure 2). Le patient a bénéficié d'un remplissage prudent, d'une transfusion lente par deux culots globulaires lors d'une séance d'hémodialyse. Le lendemain, le patient a présenté une instabilité hémodynamique, une déglobulisation avec une hémoglobine à 4g/dl, d'où la décision d'admettre le patient au bloc opératoire pour un geste d'hémostase chirurgical. Après un monitorage, le patient a été intubé, ventilé. Quelques minutes après, le patient a présenté un état de choc d'où le recours à la noradrénaline à la dose de 0,25µg/kg/min. L'exploration chirurgicale a mis en évidence un énorme hématome rétropéritonéale avec un saignement actif au niveau du rein gauche déchiqueté. Une néphrectomie d'hémostase a été réalisée (Figure 3). Le saignement peropératoire a été estimé à 1,5 litre. Le patient s'est stabilisé puis transféré ensuite au service de réanimation polyvalente. L’évolution a été marquée par une amélioration et le patient a été transféré deux jours après au service de néphrologie.

## Discussion

Quarante ans après son introduction par Iversen et Brun, la biopsie rénale percutanée garde une place privilégiée parmi les examens complémentaires en néphrologie, c'est l'examen indispensable au diagnostic histologique de la plupart des maladies rénales parenchymateuses [1]. La Ponction Biopsie Rénale (PBR) est un outil sûr et efficace dans le diagnostic et la gestion de la maladie rénale, ses résultats guident le traitement étiologique, aident à établir un pronostic rénal et permettent de mieux définir les mécanismes physiopathologiques des atteintes rénales. Deux modifications techniques majeures ont augmenté significativement sa sécurité et son efficacité: échoguidage en temps réel et l'utilisation du pistolet automatique [2]. Dans les séries récentes le taux global de complications oscille entre 13 et 34% et le taux de complications sévères entre 1,2 et 6,4% [3, 4]. Une étude de *Whittier*, de 750 biopsies sur reins natifs (>15 ans) avec une surveillance intrahospitalière de 24h, a objectivé des complications majeurs ont eu lieu dans 48 (6,4%) des patients et mineurs dans 6,6% des cas [4] dont la douleur, expliquée essentiellement par l'effraction cutanée et pariétale et surtout quand l'effet de l'anesthésie locale se dissipe d'où la prémédication avec une bonne analgésie après le geste [5], dans notre cas le patient n'a pas bénéficié d'une prémédication, geste fait sous anesthésie locale avec injection de la Lidocaine 2% après repérage échographique, l'analgésie dans les suites immédiates assurés par Néfopam + paracétamol injectable avec bonne satisfaction du malade initialement. Les principales complications de la PBR sont d'ordre hémorragique. Elles se manifestent par des hématuries et des hématomes périrénaux [6]. Les autres principales complications décrites sont surtout des complications d'ordre hémorragiques, elles se manifestent par des hématuries macroscopiques dans 1,3 à 10%, sans formation de caillot et ne duraient pas plus de trois jours d'après Parrish [7], dans notre cas les urines d'aspect clair sans notion d'hématurie macroscopique.

L'hématurie ainsi que les autres complications hémorragiques comme l'hématome péri-rénal seraient corrélées positivement à une pression artérielle élevée [8, 9] avec un risque significatif quand la pression artérielle diastolique est supérieure ou égale à 95 mmHg. Pour notre cas, après monitorage du malade avec surveillance scopique les valeurs de pression artérielle diastolique et moyenne sont restées dans les normes. La Fistule artério-veineuse est une autre complication relativement bénigne car la fermeture spontanée est observée dans plus de 95% des cas dans les deux ans qui suivent la biopsie, l'examen au doppler est actuellement meilleur pour son dépistage [9]. Les études récentes rapportent moins de 0,1% de complications vitales; néanmoins le potentiel des complications sévères percutanés restent toujours présent; l'incidence des complications hémorragiques symptomatiques est de 13% de l'ensemble des biopsies selon Carmen [2]; La fréquence des hématomes péri-rénaux varie dans la littérature en fonction de la technique d'imagerie, elle serait de 57 à 60% lorsqu'un suivi systématique des PBR est réalisé par tomodensitométrie [10, 11]. Ces hématomes sont généralement asymptomatiques. Dans notre cas l'hématome péri-rénal a été diagnostiqué sur échographie et scanner abdominal montrant un volumineux hématome péri-rénal gauche compressive diffusant au niveau de la région pelvienne homolatérale.

Le retentissement clinique de l'hémorragie dépend de l'intensité de la spoliation sanguine et du retentissement obstructif de l'hématurie sur la voie excrétrice. Une hypotension ou une baisse de l'hématocrite de plus de 10% est observée dans 5% des cas, une transfusion érythrocytaire est nécessaire dans 0 à 3,6% des cas [6, 12],dans notre étude l'hypotension de 60 à 55 de PAM avec une déglobulisation (Hb: 08g/dl - Hb: 04 g/dl) nous a imposé une transfusion prudente par 03 CG au total sur une durée de 24 H malgré l'insuffisance rénale, dans la littérature 6 à 7% des complications cliniquement significative nécessitant une intervention thérapeutique (radiologique; chirurgicale ou transfusion de culots globulaires) [2]; une néphrectomie d'hémostase est effectuée chez moins de 0,1% des patients [5], plus de 90% des complications majeures surviennent dans les 24 H qui suivent la biopsie rénale, le cas de notre patient ayant bénéficié d'une biopsie rénale avec survenue 24 H après la biopsie d'une complication hémorragique avec instabilité hémodynamique voire choc hémorragique nécessitant le geste chirurgical en urgence avec réalisation d'une néphrectomie d'hémostase avec évacuation de l'hématome retro-péritonéal.

## Conclusion

La P.B.R. échoguidée constitue une méthode d'exploration d'organe pratiquement inoffensive, précise et de haute fiabilité avec un taux de succès de 92% et un faible pourcentage de complications de 2,6% avec parfois de complications vitale le cas de notre malade avec un taux de mortalité située entre 0,06% et 0,16% selon les auteurs [13] d'où surveillance rapprochée, après réalisation de la PBR, des paramètres vitaux et de l'aspect des urines est nécessaire pendant 24 heures.
